# Imaging glucose metabolism to reveal tumor progression

**DOI:** 10.3389/fphys.2023.1103354

**Published:** 2023-02-02

**Authors:** Yiming Meng, Jing Sun, Guirong Zhang, Tao Yu, Haozhe Piao

**Affiliations:** ^1^ Central Laboratory, Liaoning Cancer Hospital & Institute, Cancer Hospital of China Medical University, Shenyang, China; ^2^ Department of Medical Image, Liaoning Cancer Hospital & Institute, Cancer Hospital of China Medical University, Shenyang, China; ^3^ Department of Neurosurgery, Liaoning Cancer Hospital & Institute, Cancer Hospital of China Medical University, Shenyang, China

**Keywords:** molecular imaging, tumor metabolism, PET, biological characteristic, aerobic glycolysis

## Abstract

**Purpose:** To analyze and review the progress of glucose metabolism-based molecular imaging in detecting tumors to guide clinicians for new management strategies.

**Summary:** When metabolic abnormalities occur, termed the Warburg effect, it simultaneously enables excessive cell proliferation and inhibits cell apoptosis. Molecular imaging technology combines molecular biology and cell probe technology to visualize, characterize, and quantify processes at cellular and subcellular levels *in vivo*. Modern instruments, including molecular biochemistry, data processing, nanotechnology, and image processing, use molecular probes to perform real-time, non-invasive imaging of molecular and cellular events in living organisms.

**Conclusion:** Molecular imaging is a non-invasive method for live detection, dynamic observation, and quantitative assessment of tumor glucose metabolism. It enables in-depth examination of the connection between the tumor microenvironment and tumor growth, providing a reliable assessment technique for scientific and clinical research. This new technique will facilitate the translation of fundamental research into clinical practice.

## Introduction

Metabolic reprogramming is a cancer characteristic in which cells are rewired to enable optimized neoplastic development (Madhavan and Nagarajan, 2020; Lin et al., 2022). Typically, differentiated cells rely on oxidative phosphorylation within mitochondria to produce the energy required for cellular processes. In contrast, most cancer cells rely on aerobic glycolysis, a phenomenon known as the “Warburg effect” (Sieow et al., 2023). Given the dramatic increase in glucose absorption during this process, glycolysis is a promising early target for cancer therapy (Rangel Rivera et al., 2021). Therefore, study of cancer metabolism is important for understanding its basic pathophysiology and clinical oncology (Karlstaedt et al., 2021). This review focuses on the processes behind the non-invasive study of glucose metabolism dysregulation in cancer.

### The critical role of aerobic glycolysis in tumor metabolism

**FIGURE 1:**
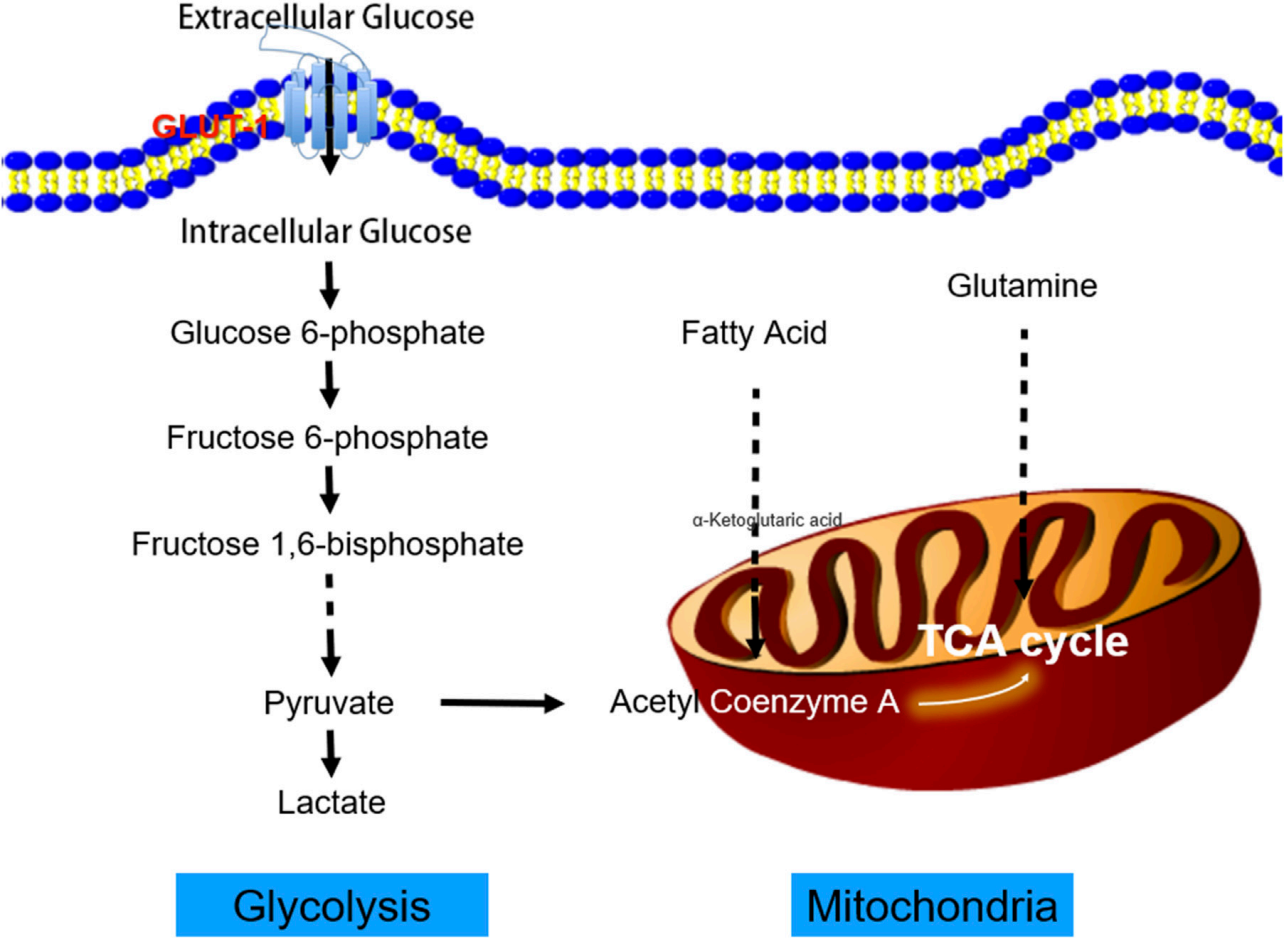
The process of tumor aerobic glycolysis. Cancer cells increase the uptake of glucose and the production of lactic acid by enhancing aerobic glycolysis. Glucose provides the primary carbon source for the tricarboxylic acid cycle (TCA). Cancer cells use the glycolytic pathway to rapidly produce adenosine triphosphate (ATP) and its intermediate product as a carbon source to synthesize macromolecular compounds, for example, to provide a sufficient carbon source for the pentose phosphate pathway to generate 5-phosphate ribose backbone. The reduced nicotinamide adenine dinucleotide phosphate (NADPH) produced by the pentose phosphate pathway can maintain the cell’s redox homeostasis. In addition, the lactic acid produced by glycolysis can promote the acidification of the tumor microenvironment, which is conducive to tumor cell invasion and migration.

A greater understanding of the interplay between systemic and tumor metabolism could uncover new treatment targets (Agbu and Carthew, 2021). Active glycolysis pathways, decreased oxygen consumption, and enhanced glucose absorption are hallmarks of solid malignant tumors. At the cellular level, glucose enters tumor cells by glucose transporters. Subsequently, it is transformed into pyruvate by a series of glycolytic enzymes. Finally, lactate dehydrogenase converts pyruvate into lactate (Guo et al., 2022; Norton et al., 2022) (Figure 1). As a proinflammatory and immunosuppressive mediator, lactic acid contributes to the malignant growth of tumors, and its accumulation in significant amounts alters the pH of the tumor microenvironment (De Jesus et al., 2022). Lactic acid can also activate matrix metalloproteinase (MMP) and directly enhance cell migration ability (Broadfield et al., 2021). In conclusion, the glucose metabolic process is helpful for tumor cell proliferation, invasion, and metastasis, as well as radiotherapy and chemotherapy resistance, which complicates treatment decisions (Zhu and Thompson, 2019; Gao et al., 2023).

### Molecular imaging enables visualization of glycolytic effects

Medical imaging techniques such as nuclear medicine, magnetic resonance imaging (MRI), and optical imaging, are at the heart of molecular imaging technology. These allow for non-invasive, real-time imaging of the cellular and molecular levels of physiological and pathological processes inside the human body (Tian et al., 2021; Hod et al., 2023). Owing to advancements in artificial intelligence and innovative imaging agents, molecular imaging may soon become an essential part of therapeutic care (Rowe and Pomper, 2022). The fluorine-18-fluorodeoxyglucose (18F-FDG) positron emission tomography (PET) is a functional molecular imaging technique that takes advantage of the enhanced glycolysis of cancer cells to obtain both structural information and metabolic activity (Vaarwerk et al., 2021). PET with innovative radiotracers and MR-based approaches provides exciting new ways to study glycolysis and broadens the metabolic imaging toolkit (Edmonds et al., 2022). To completely define tumor biology and better guide therapy, multiparametric imaging must combine various modalities.

### Principal patterns of molecular imaging to detect glycolytic effects

#### PET imaging of tumor glycolysis using pyruvate kinase M2 (PKM2)

The core of tumor metabolism is the glycolytic pathway, which promotes tumor growth through the production of ATP and synthesis of biosynthetic reaction intermediates. PKM2 catalyzes the final rate-limiting steps in tumor glycolysis and controls the balance between energy production and synthesis of metabolic precursors (Ouyang et al., 2018). Considering the importance of PKM2 in regulating tumor metabolism, scientists have attempted to measure its expression non-invasively by developing specific radioactive preparations of PKM2. Witney et al. (2015) described the manufacture and assessment of [11C]DASA-23, a PET radiotracer that offers a direct, non-invasive measurement of PKM2 expression in preclinical models of glioblastoma multiforme (GBM). This study laid the foundation for the clinical use of [11C]DASA-23, which could be used for imaging primary brain tumors and other tumors that may metastasize to the brain. Beinat et al. (2017); Beinat et al., 2020b) reported that a novel radiotracer, 1-((2-fluoro- 6-[18F]fluorophenyl)sulfonyl)-4-((4-methoxyphenyl)sulfonyl)piperazine ([18F]DASA- 23), can be safely used to evaluate pyruvate kinase M2 levels. Its feasibility and safety were further verified through animal experiments and clinical trials (Saidi et al., 2018; Beinat et al., 2021).

#### 18F-FDG-PET/CT was used to visualize the metabolic activity of surviving tumor cells

FDG is a radiolabeled glucose analog that is transported into tumor cells and phosphorylated by hexokinase (Dezhakam et al., 2022). Therefore, FDG-PET can indicate the need for glucose by cells or tumors and provide some information about the tumor’s processing of glucose into usable components (Cossu et al., 2019). Yu and his team (Liang et al., 2020) used 18F-FDG-PET/CT to visualize the metabolic activity of surviving tumor cells. The maximum standardized uptake value (SUVmax) has been used as a prognostic indicator of pancreatic ductal adenocarcinoma (PDAC). PET/CT volume parameters, metabolic tumor volume (MTV), and total lesion glycolysis (TLG) fully reveal tumor metabolic activity and volume. The transition to aerobic glycolysis is a characteristic of advanced cancers and is easily assessed by 18F-FDG-PET (Zhao et al., 2022). A crucial route governing the development and metabolism of these malignancies is the mechanistic target of the rapamycin (mTOR) pathway, which can be efficiently addressed by utilizing selective catalytic mTOR kinase inhibitors (Ferrara and Roz, 2022). This was investigated in mice with lung cancer, where therapeutic response was evaluated using 18F-FDG-PET and computed tomography (CT) imaging before and after the administration of mTOR inhibitor MLN0128 (Momcilovic et al., 2018a; Momcilovic et al., 2018b). After targeted therapy intervention, the results demonstrated that 18F-FDG-PET/CT could evaluate dynamic changes in glucose metabolism in lung cancer within mice.

#### Lactate concentration was measured by proton magnetic resonance spectroscopy (1H-MRS) and SUV by FDG-PET

**FIGURE 2:**
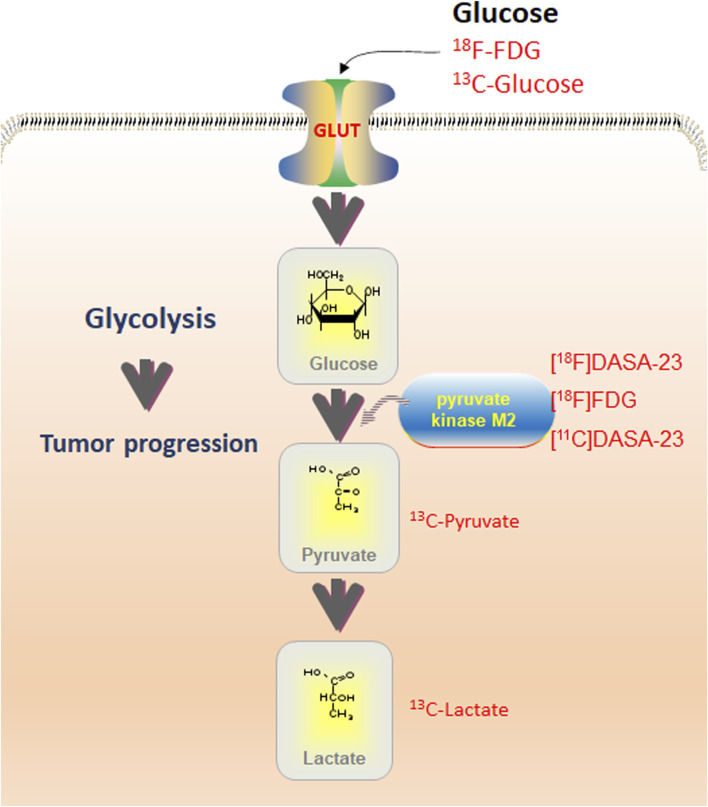
Overview of glycolysis routes detectable with various imaging techniques. PET with ^18^F-FDG may detect glucose uptake; MRS with ^13^C-Glucose can be used for optical imaging. Pyruvate kinase M2 (PKM2) is detectable with PET utilizing [^18^F]DASA-23, [^18^F]FDG, and [^11^C]DASA-23. Contribution of pyruvate and lactate may be assessed using ^13^C-Pyruvate and ^13^C-Lactate utilizing MRS. Combining imaging modalities with quantitative immunohistochemistry allows the assessment of total protein levels and phosphorylation.

PET detection of the radioactive glucose analog 18FDG is the only commonly accessible metabolic imaging technology in current clinical practice. However, 18FDG-PET findings are unclear in tissues with intrinsically high glucose absorption, such as the brain, and provides little information on the metabolic processes that occur after glucose intake (De Feyter et al., 2018; Zhang et al., 2022). 1H-MRS is a non-invasive imaging tool for brain function that can reliably detect and quantitatively evaluate brain metabolites for subjective identification of gliomas. 1H-MRS has good accuracy and specificity in diagnosis and grading, as well as the ability to assess the glioma lesion spectrum (Rodriguez-Nieto et al., 2023). This technology is also suitable for simultaneously analyzing multiple target tissue regions, thereby enabling 1H-MRS imaging (1H-MRSI). 1H-MRSI generates a “voxel” table, which can “map” lactate levels (Corbin, 2019; Lussey-Lepoutre et al., 2020). Esfahani et al. (2022) evaluated the use of hyperpolarized (HP) [1-13C]pyruvate magnetic resonance spectroscopic imaging (HP-13C MRSI) to quantitatively measure early changes in glycolytic metabolism and its ability to predict the response to pan-tyrosine kinase inhibitor (pan-TKI) therapy in gastric cancer (GC). HP-13C MRSI was found to be a more representative biomarker of early metabolic changes in response to pan-TKIs in GC than 18F-FDG-PET, and can be used for the early prediction of response to targeted therapies. The combination of FDG-PET and MRSI may provide a more sensitive clinical indicator of aerobic glycolysis (Casey et al., 2020; Tarumi et al., 2020). Therefore, an imaging technology that can identify aerobic glycolysis in tumors non-invasively would enhance cancer research and monitoring (Figure 2; Table 1 (Witney et al., 2015; Beinat et al., 2017; Momcilovic et al., 2018a; Beinat et al., 2018; Momcilovic et al., 2018b; Choi et al., 2018; Hundshammer et al., 2018; Saidi et al., 2018; Scroggins et al., 2018; Zhao et al., 2018; Beinat et al., 2020a; Beinat et al., 2020b; Yamamoto et al., 2020; Beinat et al., 2021; Esfahani et al., 2022)).

**TABLE 1:** Summary of glycolysis by molecular imaging.

Target	Imaging modality	Radiotracer	Model	Disease model/Patient type	Preclinical/Clinical	Comments	Ref
PKM2	GE PETtrace cyclotron	[^18^F]DASA-23	Cell line (HeLa cells)	Cervical cancer	Preclinical	They identified an F-18-labeled PKM2-specific radiotracer which shows potential for *in vivo* glycolysis imaging	Beinat et al. (2017)
PKM2	GE PETtrace cyclotron	[^18^F]DASA-23 and [^18^F]FDG	Cell line (U87 cells)	Glioblastoma multiforme (GBM)	Preclinical	Evaluation of glycolytic response to multiple classes of anti-glioblastoma drugs	Beinat et al. (2020b)
PKM2	PET/CT and PET/MRI	[^18^F]DASA-23	Cell line, Mouse and human	GBM	Preclinical	These findings suggest the future clinical study of [^18^F]DASA-23 for imaging therapy-induced normalization of cancer metabolism	Beinat et al. (2021)
PKM2	Cobra II Auto-Gamma Counter	[^18^F]DASA-23	Cell line	GBM	Preclinical	This research has shown that tumor-treating fields inhibit hGBM abnormal glycolytic metabolism through decreased PKM2 expression, which can be measured non-invasively by the [^18^F]DASA-23 radiotracer	Saidi et al. (2018)
PKM2	PET	[^11^C]DASA-23	Nude mice	GBM	Preclinical	These data provide imaging agents that target this critical gatekeeper PKM2 of tumor glycolysis	Witney et al. (2015)
PKM2	PET/MRI	[^18^F]DASA-23	Human	Five healthy volunteers	Clinical Trial (NCT03539731)	Their results indicate that [^18^F]DASA-23 can be used safely in humans to evaluate pyruvate kinase M2 levels	Beinat et al. (2020a)
PKM2	PET	[^18^F]DASA-23 *via* fluorination of 1-((2-fluoro-6-nitrophenyl)sulfonyl)-4-((4-methoxyphenyl)sulfonyl)piperazine with K [^18^F]F/K2.2.2	Mice	Prostate cancer	Preclinical	They observed rapid metabolism of [^18^F]DASA-23 in mouse plasma	Beinat et al. (2018)
PKM2	PET/CT	conventional parameters, total lesion glycolysis, and heterogeneity parameters	Nude mice	HR^+^HER2^-^ metastatic breast cancer	Preclinical	^18^F-FDG based intra-tumor heterogeneity appears to be a potential predictor of the efficacy of fulvestrant among HR^+^HER2^-^ metastatic breast cancer patients	Zhao et al. (2018)
^18^F-FDG	PET/CT	standard uptake values (SUV)	Genetically engineered mouse models	Lung cancer	Preclinical	^18^F-FDG PET/CT can be used to evaluate dynamic changes in glucose metabolism in lung cancer mice after the targeted therapy intervention	Momcilovic et al. (2018a), Momcilovic et al. (2018b)
^18^F-FDG	new hyperpolarized MRI and EPR imaging procedures	pO_2_ and^18^F-FDG uptake, lactate dehydrogenase activity	Nude mice	Pancreatic tumor	Preclinical	They developed a novel multimodal molecular imaging technique to reveal three companion imaging biomarkers and describe the relationship between hypoxia, glucose uptake, and glycolysis in the tumor microenvironment	Yamamoto et al. (2020)
^18^F-FDG and^13^C-pyruvate	PET/MR	SUV and apparent diffusion coefficients (ADC)	Rats	Breast cancer	Preclinical	They concluded that changes in cell density affect PET and^13^C data in a similar way. The correlation of longitudinal metabolic data seems to reflect biochemical processes and tumor cell structure	Hundshammer et al. (2018)
Hyperpolarized Sodium [1- ^13^C]-Glycerate	Magnetic resonance spectroscopy (MRS)	[^13^C]-labeled pyruvate and lactate	Rats	Healthy male	Preclinical	This study established a hyperpolarized [^13^C]-Glyc, which provides an opportunity to evaluate the redox state of cells in biochemical research	Choi et al. (2018)
Hyperpolarized [1-^13^C]-pyruvate	Magnetic resonance spectroscopic imaging (MRSI)	[1-^13^C]-pyruvate	Mouse	Prostate cancer	Preclinical	Hyperpolarized [1-^13^C]-pyruvate MRSI of prostate cancer predicts the efficacy of targeting the Warburg effect	Scroggins et al. (2018)
Hyperpolarized [1-^13^C]Pyruvate	MRSI	Hyperpolarized [1-^13^C]Pyruvate	Mouse	Gastric cancer (GC)	Preclinical	HP-^13^C MRSI is a more representative indicator of early metabolic alterations in response to pan-TKI in GC than [^18^F]FDG PET.	Esfahani et al. (2022)

### Molecular imaging technology combined with glycolysis products

#### FDG-PET in conjunction with HP MRI

HP MRI provides additional information that cannot be inferred from FDG-PET or hypoxia measures (Zhao et al., 2018). The tissue contrast of 18F-FDG-PET in the prostate is poor and its application is complicated because of its proximity to the excretory system. In addition, the fundamental change in prostate cancer is a change in carbon use, not just glucose uptake. Therefore, after the injection of HP [1-13C] pyruvate, rapid 13C MR was used to measure the metabolic conversion of this aggressive cancer and its response to treatment, with direct biological significance (Fantin et al., 2006; Keshari et al., 2013a; Keshari et al., 2013b; Wilson and Kurhanewicz, 2014). Using this approach, Qi et al. (2022) utilized 18F-FDG-PET and HP [1-13C]pyruvate MRSI with chemical exchange saturation transfer (CEST) MRI measures to investigate the effect of glucose infusion on intracellular pH and its relationship to the tumor.

#### Chemokine receptor-4 targeted PET/CT with 68Ga-Pentixafor

While 18F-FDG-PET/CT glucose absorption measurements provides useful metabolic information for evaluating most malignant tumors it is limited in evaluating multiple myeloma (MM) (Zamagni et al., 2021). Since chemokine receptor four is overexpressed in MM, Pan and his team (Pan et al., 2020) conducted a prospective cohort study to compare the performance of 68Ga-pentixafor and 18F-FDG-PET/CT in newly diagnosed MM. Quantitative analysis revealed that the bone marrow uptake value of 68Ga-Pentixafor (TBmUCXCR4, SUVmax, and SUVmean) was positively correlated with tumor burden-related end-organ damage, and the stage was positively associated with laboratory biomarkers. 68Ga-Pentixafor was more abundant than 18F-FDG in newly diagnosed MM. These results indicated that 68Ga-pentixafor might be a superior biomarker to 18F-FDG for PET quantification assessment of tumor burden in newly diagnosed MM. However, further investigations are needed to determine whether this affects patient prognosis and survival.

#### Combined molecular imaging of glycolysis with angiogenesis

Clemmensen et al. (2021) used [68Ga] Ga-NODAGA-RGD-PET with vascular imaging and hyperpolarization [1-13C] pyruvate-MRSI to detect energy metabolism. [68Ga] Ga-NODAGA-RGD-PET and [1-13C] pyruvate-MRSI may provide additional information than either technique alone, indicating that non-invasive hyperPET combined with angiogenesis and glycolysis imaging can help to analyze cancer phenotypes. They showed that imaging of angiogenesis and tumor metabolism by [68Ga]Ga-NODAGA-RGD-PET and [1-13C] pyruvate-MRSI, respectively, is feasible in canine cancer patients.

#### Synergistic construction of aerobic and anaerobic glycolysis zones

As reported, tumor hypoxia and glycolysis, including low-oxygen glycolysis and anaerobic glycolysis, were predicted using 18F-FDG and 18F-fluoromisonidazole (FMISO) PET technologies (Leimgruber et al., 2020). This method has been applied in a prospective clinical trial of 10 patients with GBM who underwent 18F-FDG and 18F-FMISO PET and MRI studies after surgery, before radiotherapy, or early after radiotherapy. Spatial mapping of aerobic and anaerobic glycolysis can provide unique information regarding tumor metabolism and hypoxia through PET. This prospective preliminary study will facilitate the development of a powerful and intuitive software tool for assessing glucose metabolism under hypoxic conditions. In addition, a unique software algorithm, “Glyoxia,” has been developed (Bi et al., 2019; Moscoso et al., 2019), which can detect glycolysis and hypoxia through the automatic processing of 18F-FDG and 18F-FMISO PET images. The algorithm allows serial dual-track molecular imaging comparisons to classify and delineate hypoxic areas at a risk of recurrence. The simplicity of this method can be extended to other hypoxic cancers and possibly to other molecular imaging compounds. By understanding the glucose metabolism in GBM under hypoxic conditions, the effects of aerobic glycolysis in patients and the relationship with treatment resistance can be assessed.

### A new hybrid mode—PET/MR

Precision treatment is inseparable from the development of biomedical imaging technology, aiming to identify changes in tumor biology at the cellular and molecular levels (Goutsouliak et al., 2020). The novel PET/MR hybrid imaging modality allows for the simultaneous acquisition of high-resolution anatomical images and metabolic data. The critical step of the Warburg effect, a hallmark of tumors, can be measured non-invasively using this emerging technology. Hundshammer and his team (Hundshammer et al., 2018) utilized the multimodal imaging workflow of the PET/MR system, including proton MRI, to obtain accurate morphological information and diffusion-weighted imaging (DWI) to resolve tumor cellularity. Metabolic data were measured using dynamic 18F-FDG-PET and HP 13C-pyruvate MRSI. This technique was also used to determine how cell density influences variations in glycolytic parameters that occur due to cell expansion. Researchers have found that an increase in LDH activity corresponded with increased glucose absorption by tumor cells. Longitudinal DWI data showed that cell density decreased during tumor growth, affecting the quantification of PET and MRSI data. The workflow included multiparameter and non-invasive tumor characterization, which can be applied to larger animal models. This has the potential for clinical applications, paving the way for tailored and patient-specific treatment methods.

### Visualization of glucose metabolism for cancer immunotherapy

Immunotherapy replaces traditional radiotherapies and chemotherapies with immune checkpoint inhibitors, which use the patient’s immune system to identify and target cancer cells (Vernieri et al., 2022). Despite the tremendous clinical success and aggressive research on immunotherapies, there remains a considerable unmet need for a rigorous technique to identify immunotherapy responders (Kraehenbuehl et al., 2022). Early and precise monitoring of immunotherapy response is essential for optimal medication development and tailored treatment (Goncalves et al., 2021). Texture characteristics generated from PET, which are known to suggest glucose absorption distribution, have been shown to be linked with the expression of PD-L1 mRNA, but not with the expression of PD-1, CTLA-4, or TMB. To differentiate between those who will respond to treatment and those who will not, non-invasive *in vivo* 18F-FDG-PET/CT imaging of glucose metabolism in lymphatic organs might be a valuable preclinical and clinical technique (Schwenck et al., 2020). Vos et al. (2022) discovered 18F-FDG-PET-based DeltaMTV and DeltaTLG reliably identified pathological responses to neoadjuvant immune checkpoint blockade (ICB) in head and neck squamous cell carcinoma (HNSCC) before surgery, surpassing the European Organisation For Research And Treatment Of Cancer (EORTC) criteria. However, neck lymph nodes exhibited pseudoprogression. Upon validation, 18F-FDG-PET might identify HNSCC patients for response-driven therapy adaptation in future clinical studies. PET biomarkers based on 18F-FDG imaging have been successfully used for prognosis and therapeutic response monitoring in patients undergoing anti-PD1 immunotherapy for malignant melanoma, where hematopoietic tissue metabolism correlates adversely with survival. Additionally, compared to CT imaging alone, metabolic imaging with FDG-PET at termination of immune checkpoint inhibitor medication helps identify melanoma patients with a low risk of recurrence and favorable prognosis (Ferdinandus et al., 2022). Yang’s group (Yang et al., 2022) demonstrated the promise of sensitive and comprehensive monitoring using label-free metabolic intravital imaging (LMII) for visualizing the dynamic changes in heterogeneous cell metabolism of cancer cells and immunological infiltrates in response to immunotherapy. The uptake of 18F-FDG and 18F-fluorodeoxythymidine (FLT) on PET may serve as an imaging biomarker for assessing the efficacy of immune checkpoint inhibitor therapy (Oh et al., 2022). Another research group (Wang Y. et al., 2020) looked at how the expression of intra-tumor immunomarkers in non-small cell lung carcinoma (NSCLC) patients correlated with their metabolic status, as measured by 18F-FDG-PET/CT. These findings showed that SUVmax on 18F-FDG-PET/CT may be a valuable predictor for identifying patients for immunotherapy by revealing a link between metabolic variables and immune cell expression in the tumor microenvironment. In oral squamous cell carcinoma, the absorption of 18F-FDG is an independent predictor of cold tumors. Imaging using 18F-FDG-PET may be a viable diagnostic technique for estimating the immunological state of tumors. However, quantitative 18F-FDG-PET imaging of the spleen performed poorly in predicting clinical improvement using ipilimumab (Sachpekidis et al., 2019).

### Importance in clinical practice

By leveraging the radiological properties of SUVmean and entropy on 18F-FDG-PET/CT, researchers discovered a potential benefit of 2-year progression-free survival (PFS) (Mayerhoefer et al., 2019). Moreover, when combined with clinical/laboratory and biological parameters, they could obtain more valuable PFS and prognostic information. Ceriani and his team (Ceriani et al., 2021) established a multiparameter prediction model that integrated texture features and conventional PET indicators into incidental thyroid tumors. This model can be combined with three independent PET-derived prediction parameters: TLG, SUVmax, and shape sphericity. This approach was used in a study monitoring 19 patients with malignant pleural mesothelioma (MPM) before and after two cycles (6 weeks) of chemoimmunotherapy (Reynolds et al., 2020). To determine the malignancy of [18F]-fluorodeoxyglucose-avid thyroid incidentalomas, six separate radiological characteristics were used that describe the shape, heterogeneity, and intensity of lesion tracer uptake. A threshold-based Interactive Data Language (IDL) tool was then created to quantify tumor volume in 18F-FDG-PET scans. In this MPM study, there was a causal relationship between tumor volume and TLG. Research also showed that, regardless of whether the tumor size changed or responded to therapy, the overall intake of 18F-FDG is always proportional to the total metabolite volume (TMV) of the tumor. This finding was unexpected because standard teaching indicates that changes in metabolism, measured by FDG uptake, precede changes in volume. MPM may be a condition in which FDG uptake by each cell and its proliferation vary simultaneously. Additional clinical data are listed in Table 2 (Kahraman et al., 2012; Lee et al., 2013; Maharjan et al., 2013; Usmanij et al., 2013; Bhoil et al., 2014; Boers-Sonderen et al., 2014; Choi et al., 2014; Soussan et al., 2014; Elimova et al., 2015; Tateishi et al., 2015; Wong et al., 2016; Xie et al., 2016; Lim et al., 2017; Liu et al., 2017; Park et al., 2017; Salavati et al., 2017; Calandriello et al., 2018; Kaira et al., 2018; Lovinfosse et al., 2018; Wang et al., 2018; Avallone et al., 2019; Carpenter et al., 2019; Dag et al., 2019; Mayerhoefer et al., 2019; van Berkel et al., 2019; Wang L. et al., 2020; Deckers et al., 2020; Fiore et al., 2020; Mathew et al., 2020; Mitchell et al., 2020; Pan et al., 2020; Dunet et al., 2021; Vos et al., 2022).

**TABLE 2:** Clinical trials of glycolysis by FDG molecular imaging.

Imaging modality	Radiotracer	Predicator	Disease model/Patient type	Clinical application value	Ref
PET/CT	^18^F-FDG	SUVmax^a^, MTV^b^ and TLG^c^	Advanced biliary tract cancer (BTC)	MTV may be considered an essential independent metabolic prognostic factor for the overall survival of patients with BTC, and it is a predictive marker for erlotinib treatment	Choi et al. (2014)
PET/CT	^18^F-FDG	Tumor diameter, SUVmax, and TLG	Advanced gastric cancer (AGC)	Both tumor size and SUVmax are related to the patient’s OS and PFS, which can help predict the prognoses of AGC patients receiving chemotherapy	Park et al. (2017)
PET/CT and DW MRI	^18^F-FDG	Tumor volume or TLG	Advanced head and neck squamous cell carcinoma (HNSCC)	The data indicate that the metabolic tumor volume or TLG caused by^18^F-FDG PET/CT obtained after one cycle of induction chemotherapy is an early predictive biomarker for the final response to subsequent radiotherapy and chemotherapy	Wong et al. (2016)
PET/CT	^18^F-FDG	Median ΔSUV_max_, ΔSUV_mean_, ΔMTV, and ΔTLG	HNSCC	Assessment of primary tumor responses to neoadjuvant immune checkpoint blockade in HNSCC using [^18^F]FDG-PET-based MTV and TLG reliably detects pathological responses early in treatment	Vos et al. (2022)
PET/CT	^18^F-FDG	SUV^d^, MTV and TLG	Breast, endometrial, and ovarian cancer	Early response assessment with FDG-PET/CT can predict local remission (PR) and progression (PD)	Boers-Sonderen et al. (2014)
PET/CT	^18^F-FDG	SUVmax, SUVmean, MTV and TLG	Cervical cancer	Among high-risk cervical cancer patients receiving mid-chemoradiation therapy (CRT) and brachytherapy, the TLG and metabolic tumor volume of FDG-PET/CT in CRT are related to OS. These indicators may provide early signals for enhancing dose escalation or selective treatment of adjuvant chemotherapy	Carpenter et al. (2019)
PET/CT	^18^F-FDG	SUVmax, MTV and TLG	Cervical cancer	This study introduces the metabolic parameters of primary tumors and regional lymph nodes measured by F-18 FDG PET/CT pretreatment. The SUVmax of CT pretreatment may be a prognostic biomarker for predicting the recurrence of locally advanced cervical cancer	Dag et al. (2019)
PET/CT	^18^F-FDG	SUVmax, MTV and TLG	Diffuse large B-cell lymphoma	This study found that the SUVmax value cannot predict PFS, and MTV and TLG may be critical prognostic markers of PFS in DLBCL.	Xie et al. (2016)
PET/CT	^18^F-FDG	SUV_max_, SUV_mean_ ^e^ and TLG	High-Risk Locally Advanced Rectal Cancer	Early total-lesion glycolysis and its percentage change compared with baseline (ΔTLG-early) showed the highest accuracy in predicting the regression of advanced rectal cancer. It is possible to guide the decision to modify the treatment plan during preoperative radiotherapy and chemotherapy based on the particular response	Avallone et al. (2019)
PET/CT	^18^F-FDG	TLG	Locally advanced rectal cancer (LARC)	This study found that TLG of the primary tumor in FDG-PET/CT can be considered a prognostic factor for locally advanced rectal cancer patients receiving neoadjuvant chemoradiotherapy (CCRT)	Lee et al. (2013)
PET/CT	^18^F-FDG	SUVmax, SUVmean, MTV, TLG, and histogram-intensity features	LARC	The histological analysis of baseline^18^F-FDG PET/CT can be a strong independent predictor for the survival of LARC patients	Lovinfosse et al. (2018)
PET/CT	^18^F-FDG	SUVmean	Mantle cell lymphoma (MCL)	SUVmean and entropy can improve the prediction of PFS and PFS prognosis in 2 years. When metabolic indicators are combined with clinical, laboratory, and biological parameters, the best predictive analysis results can be achieved	Mayerhoefer et al. (2019)
PET/CT	^18^F-FDG	TLG	Metastatic colorectal cancer	Measurement of TLG can predict the treatment outcome of regorafenib in mCRC.	Lim et al. (2017)
PET/CT	^68^Ga-Pentixafor and ^18^F-FDG	Total bone marrow glycolysis, whole bone marrow uptake, total bone marrow volume [TBmV], SUVmean and SUVmax	Multiple myeloma	^68^Ga-Pentixafor PET/CT is expected to evaluate newly diagnosed MM.	Pan et al. (2020)
PET/CT	F-FDG and F-DOPA	SUVmax, SUV threshold, MTV, TLG, dopaminergic tumor volume (DTV), and total lesion F-DOPA activity (TLDA)	Neuroblastoma	Both F-DOPA and F-FDG PET can be used for neuroblastoma risk stratification. The correlation between the volume index of F-DOPA and F-FDG PET and the risk group is higher	Liu et al. (2017)
PET/CT	FDG	MTV, TLG, SUVmax, SUVmean, partial-volume-corrected TLG (pvcTLG), and pvcSUVmean	Non-small cell lung cancer (NSCLC)	FDG-PET/CT parameters, including MTV, TLG, and pvcTLG, have a vital prognosis for OS in patients with locally advanced non-small cell lung cancer and have similar discrimination capabilities	Salavati et al. (2017)
CT and PET/CT	FDG	blood flow (BF), blood volume (BV), mean transit time, and peak enhancement intensity. SUVmax, SUVpeak, SUVmean, MTV and TLG	NSCLC	The larger the tumor, the lower the BF and BV; conversely, the higher the SUV_peak_, MTV, and TLG. This index can be used for clinical diagnosis or treatment of NSCLC.	Calandriello et al. (2018)
PET/CT	3′-deoxy3′-fluoro-18-fluorothymidine (^18^F-FLT) and^18^F-FDG	speak values and TLG	NSCLC	This study used semi-quantitative analysis to calculate the standard intake lean body mass (SUL_peak_) and TLG values ​​of the hottest lesions in all patients. Among responders and non-responders, EGFR^18^F -FDG SUL_peak_ is better than^18^F-FLT SUL_peak_ in predicting OS and PFS in NSCLC patients treated with EGFR-TKI for 3 weeks	Bhoil et al. (2014)
PET/CT	^18^F-FDG	SUVmax, SUVmean, MTV and TLG	NSCLC	This study points out that in locally advanced NSCLC, the ratio of MTV to TLG is the only indicator related to the survival of the tumor after induction chemotherapy	Soussan et al. (2014)
PET/CT	^18^F-FDG	SUV, MTV, and TLG	NSCLC	This study proved that the use of molecular imaging to analyze the degree of change in TLG could predict the response of NSCLC to radiotherapy, chemotherapy, and PFS.	Usmanij et al. (2013)
PET/CT	^18^F-FDG	TLG and tumor lesion proliferation (TLP)	NSCLC	This study found that in patients with advanced NSCLC, the percentage change of TLG and TLP and absolute residual TLG and TLP levels under erlotinib treatment have become essential predictors of PFS.	Kahraman et al. (2012)
PET/CT	^18^F-FDG	SUVmax, MTV and TLG	NSCLC	Metabolic response by^18^F-FDG effectively predicted efficacy and survival 1 month after nivolumab treatment	Kaira et al. (2018)
PET/CT	^18^F-FDG	SUVmax, SUVtotal, SUVmean and TLG	NSCLC	Studies have proven that^18^F-FDG SUVmax has potential value as a non-invasive clinical indicator of tumor immune metabolism phenotype in patients with resectable NSCLC and can be used as a potential predictor of response to treatment based on immunotherapy strategies	Mitchell et al. (2020)
PET/CT	^18^F-FDG	SUVmean, SUVmax, MTV and TLG	NSCLC	The study found that in untreated stage IIIB-IV NSCLC, the use of pSUVmax on^18^F-FDG PET/CT may be a potential biomarker for pPDL1 TPS <1%, 1%–49%, and ≥50%, and may help determine the immunotherapy strategy for advanced NSCLC.	(Wang et al., 2020)
PET/CT	^18^F-FDG	SUVmax and TLG	Esophageal cancer	This study found that PET parameters cannot predict complete pathological remission (pathCR) in patients with esophageal adenocarcinoma or squamous cell carcinoma suitable for triple therapy. Still, baseline and TLG after mid-term or chemical radiation (CTRT) can predict OS.	Elimova et al. (2015)
PET/CT	^18^F-FDG	SUVmax, SUVmean, MTV, TLG, mean and minimum apparent diffusion coefficient (ADCmean and ADCmin), diffusion total volume (DTV), and MTV/ADCmin ratio	Pancreatic ductal adenocarcinoma (PDAC)	MTV is an independent predictor of OS and DSS, while DTV is an independent predictor of PFS. The study also found that ADC and SUV values are related and, combined with PET-MRI indicators, can help predict PDAC grade and patient survival	Dunet et al. (2021)
PET/CT	^18^F-FDG	SUVmax, MTV and TLG	Locally advanced pancreatic cancer	Under baseline^18^F-FDG PET/CT and its combination, the level of CA19-9 before treatment and the MTV and TLG values ​​of the primary tumor may predict early progression (EP), local progression (LP), and overall survival (OS) in LAPC patients	Fiore et al. (2020)
PET/CT	^18^F-FDG	SUVmean, SUVmax, MTV and TLG	Pediatric anaplastic large cell lymphoma (ALCL)	Using MTV indicators for systemic tumor burden assessment and mid-term response may help ALCL patients who benefit from intensive treatment	Mathew et al. (2020)
Dynamic PET/CT	^18^F-FDG	Glucose metabolic rate (MRglc) and SUVmax of the lesions	Pheochromocytomas and paragangliomas (PPGL)	Dynamic PET/CT scans to assess the pharmacokinetics of^18^F-FDG can perform *in vivo* metabolic tumor analysis in patients with PPGL, and cluster 1 PPGL can be identified	van Berkel et al. (2019)
PET/CT	^18^F-FDG	SUVmax, MTV and TLG	Recurrent carcinoma of the cervix	This study found that SUVmax and distant metastasis on F-FDG PET-CT were independent predictors of PFS and OS in patients with recurrent cervical cancer	Maharjan et al. (2013)
PET/CT	^18^F-FDG	SUVmax, SUL, MTV, TLG, and AUC-CSH of the lesion	Relapsed or refractory diffuse large B-cell lymphoma (DLBCL)	The total lesion glycolysis calculated by PET/CT can be used to quantify the response to DLBCL treatment. It can also predict the progression-free survival of patients with relapsed or refractory DLBCL treated with bendamustine-rituximab after the last treatment cycle	Tateishi et al. (2015)
PET/CT	^18^F-FDG	MATV, SUVmean/max and TLG in contrast to LDH	Stage IV melanoma	This study evaluated the association between biomarkers (S-100B, LDH) in stage IV melanoma and PET-derived indicators SUVmean/max, metabolically active tumor volume (MATV), and TLG.	Deckers et al. (2020)
PET/CT	^18^F-FDG and^68^Ga-NOTA-PRGD2	SUV, MTV, and TLG	Thyroid cancer	The combined application of^18^F-FDG and^68^Ga-NOTA-PRGD2 PET/CT can predict and evaluate early apatinib-treated radioactive iodine-refractory thyroid cancer	Wang et al. (2018)

^a^ SUVmax: the maximum standardized uptake value.

^b^ MTV: metabolic tumor volume.

^c^ TLG: total-lesion glycolysis.

^d^ SUV: standardized uptake value.

^e^ SUVmean: the average SUVs in the regions of interest.

Through different molecular imaging technologies, the movement of cells can be tracked to determine the dynamic process of aerobic glycolysis. Molecular imaging enables researchers to observe cellular processes in their natural environment in real time, thereby significantly increasing the value and authenticity of the observations (Yamamoto et al., 2020; Chen et al., 2021; Goncalves et al., 2021). Cancer cells divide rapidly as they expand and require enormous amounts of glucose for their high metabolic rates. As a result, tumor cells *in vitro* will incorporate a large amount of 18F-FDG. Positrons emitted by 18F can be captured and visualized by the PET/CT detector, showing the location, shape, size, number, and distribution of glucose metabolism in the tumor (Nakamoto et al., 2021). This method efficiently evaluates alterations in the tumor’s energy metabolism region, judgement of benign/malignant status, staging of the lesion, and survival status of tumor cells after radiation or chemotherapy (Gomes et al., 2017; Ayubcha et al., 2021). PET/CT hypoxia imaging can detect the hypoxia status in tumor tissues *in vivo* using molecular probes. Hypoxia imaging agents such as 18F- FMISO, 18F-flortanidazole (HX)4, 18F-Fluoroazomycin Arabinoside (FAZA), and 64Cu-ATSM, have been exploited to effectively demonstrate the hypoxic status inside tumors (Ayati et al., 2021).

18F-FDG-PET/CT was shown to have 100% sensitivity and 78% specificity in monitoring mediastinal lymph node metastasis of NSCLC, according to a study published by Jimenez-Bonilla et al. (2016). Recently, Chinese researchers (Kang et al., 2016) performed 68Ga-labeled RGD peptide-alpha peptide II (68Ga-alpha II) and 18F-FDG-PET/CT imaging in patients with NSCLC and tuberculosis (TB), respectively, and 68Ga-alpha II imaging SUVmax and SUVmean. In detecting NSCLC metastatic lymph nodes, 68Ga-Alfatide II was more specific but less sensitive than 18F-FDG; the false positive rate of 68Ga-Alfatide for TB lymph node identification was much lower than that of 18F-FDG. These findings also indicate that FDG-PET may be used with other standard diagnostic procedures, such as breast ultrasonography and mammography, to further improve diagnostic accuracy. More clinical data are required to support the use of FDG-PET for evaluating lymph node metastases in the context of diagnosis (Stewart et al., 2018; Fowler and Strigel, 2022).

## Conclusion

Aerobic glycolysis is a crucial characteristic of tumors and is intimately connected to the incidence and aggressive growth of tumors (Xia et al., 2021). Monitoring aerobic glycolysis in tumor tissue might lead to improved methods for diagnosing and treating cancer (Ma et al., 2022; Raskov et al., 2022). Molecular imaging is an emerging interdisciplinary subject in imaging, medicine, and molecular biology (Jan De Beur et al., 2022). Early detection, tailored treatment, and real-time tumor monitoring will benefit from non-invasive molecular imaging of glucose metabolism in cancer. However, current technology for molecular imaging has deficiencies in terms of resolution, detection limit, accessibility, and energy extension (Ambrosini et al., 2021). Thus, combining many imaging methods may broaden the possibilities of molecular imaging (Lewis et al., 2021). Future multimodal imaging must focus on safe, effective, dual-functional molecular detection and therapy of glucose metabolism to enable the widespread uptake of molecular imaging technologies.
